# SawitMVC: A multi-view oil palm fruit bunch dataset for detection and counting

**DOI:** 10.1016/j.dib.2026.112990

**Published:** 2026-06-20

**Authors:** Fatma Indriani, Setyo Wahyu Saputro, Muhammad Zainal Muttaqin, Alia Rahmi, Triando Hamonangan Saragih, Rahmat Budianoor, Hartoni, Dwi Kartini, Naufal Said

**Affiliations:** a Department of Computer Science, Faculty of Mathematics and Natural Sciences, Lambung Mangkurat University, Banjarbaru, Indonesia; b Department of Agro-Industrial Technology, Faculty of Agriculture, Lambung Mangkurat University, Banjarmasin, Indonesia; c Department of Agribusiness, Faculty of Agriculture, Lambung Mangkurat University, Banjarmasin, Indonesia

**Keywords:** Maturity classification, Cross-angle deduplication, Tree-level ground truth, YOLO annotation, *Elaeis guineensis*, Precision agriculture, Black bunch census

## Abstract

Oil palm (*Elaeis guineensis*) plantation yield is estimated periodically through the Black Bunch Census (BBC), a field practice in which trained staff count and classify fruit bunches to project output for the coming months. Photographing each tree from multiple positions captures all visible bunches but introduces a cross-view deduplication problem: the same bunch appears in several images, and naive summation of detections far exceeds the true unique count. No publicly available oil palm dataset addresses this problem with annotated ground truth. SawitMVC contains 3992 smartphone images of 953 oil palm trees collected from two commercial plantations in Kabupaten Tanah Laut, Kalimantan, Indonesia (DAMIMAS plantation: 854 trees; LONSUM plantation: 99 trees), each photographed from four sides at 90-degree intervals or eight sides at 45-degree intervals. Bunches are annotated across four BBC maturity classes by estimated months until harvest: B1 (≤1 month), B2 (∼2 months), B3 (∼3 months), and B4 (∼4 months), in YOLO bounding box format for detection and classification tasks. A separate per-tree JSON ground truth layer records 9823 unique bunches with their cross-view appearance links, supporting direct evaluation of counting algorithms independently of detection performance.

Specifications Table

**Table:** 

Subject	Computer Sciences
Specific subject area	Computer vision; Agricultural engineering; Oil palm cultivation
Type of data	Images (.jpg); text files (.txt, YOLO bounding box labels); JSON files (per-tree ground truth); Parquet file (ground truth summary table)
Data collection	953 oil palm trees collected from two commercial plantations in Kalimantan, Indonesia (DAMIMAS plantation: 854 trees; LONSUM plantation: 99 trees). Photographed from four or eight positions around each trunk at equal angular intervals using consumer smartphones; images stored at 960 × 1280 pixels in portrait JPEG. Data collected February 2026. Bounding box and maturity class labels (four classes: B1–B4) applied by trained annotators under expert agronomist supervision. Cross-view bunch identity links annotated using a custom web-based tool.
Data source location	Country: Indonesia. City/Region: Kabupaten Tanah Laut, Kalimantan Selatan. Institution: Universitas Lambung Mangkurat (ULM), Banjarmasin, Indonesia.
Data accessibility	The data is publicly available. Repository name: Zenodo Data identification number: 10.5281/zenodo.20336322 Direct URL to data: https://zenodo.org/records/20336323
Related research article	None

## 1 Value of the Data

• SawitMVC is the first public oil palm dataset that pairs per-image bounding box annotations with per-tree ground truth for unique bunch counts, supporting independent benchmarking of detection and counting methods against separate evaluation targets.
• The dataset provides two annotation layers: YOLO bounding box labels across four maturity classes (B1 (≤1 month), B2 (∼2 months), B3 (∼3 months), and B4 (∼4 months) to harvest) for detection and classification tasks, and per-tree JSON ground truth files recording 9823 unique bunches with their cross-view appearance links for counting evaluation. The two layers can be used independently or jointly.
• Each of the 953 trees is photographed from four to eight fixed positions at approximately 90- or 45-degree intervals. Cross-view bunch appearance records in the JSON ground truth provide the supervision needed to develop and evaluate multi-view bunch deduplication algorithms.
• The dataset covers two commercial oil palm varieties, DAMIMAS (DxP Dami Mas, 854 trees) and LONSUM (PT PP London Sumatra Indonesia, 99 trees), acquired under real plantation conditions in Kalimantan Selatan, Indonesia using ten different consumer smartphone models. A pre-defined train, validation, and test split supports reproducible benchmarking.
• The dataset is openly accessible on Zenodo under the Creative Commons Attribution-NonCommercial 4.0 International licence, with a queryable Parquet summary table and ML Croissant metadata. It is broadly applicable to researchers in computer vision, agricultural engineering, and precision agriculture.

## 2 Background

Oil palm (*Elaeis guineensis*) accounts for approximately 40% of current global annual demand for vegetable oil as food, animal feed, and fuel, while planted oil palm occupies less than 5.5% of total global oil-crop area [1]. Plantation yield is estimated periodically through the Black Bunch Census (BBC), a non-destructive field practice in which trained staff count and classify visible fruit bunches across a sample of trees to project aggregate output for the coming months [2], preferred over destructive alternatives such as Oil Palm Dissection [3] for its operational practicality and forecasting horizon. The BBC classifies bunches into four stages by estimated months until harvest: B1 (≤1 month), B2 (∼2 months), B3 (∼3 months), and B4 (∼4 months). Each class maps to a monthly ripening fraction in the Ulu Bernam yield forecasting model [2,4], making per-class bunch counts the primary operational output rather than a total count alone.

Oil palm fruit bunches emerge at varying heights around the full circumference of the canopy, so no single viewpoint captures the complete bunch inventory of a tree. Circumferential multi-view imaging and three-dimensional sensing are both practical approaches to this geometry, but each introduces the same counting challenge: the same physical bunch may be observed from multiple positions and must be counted only once. Multi-view photography from fixed positions around the trunk addresses this geometry directly while remaining compatible with consumer smartphones and existing field workflows, but naive aggregation of per-view detections overcounts the true unique bunch total without cross-view deduplication.

Existing public oil palm datasets address detection and classification but do not provide a per-tree count of unique bunches that is independent of detection output [[5], [6], [7]]. Multi-view counting datasets for other fruit crops demonstrate that separating counting ground truth from detection labels is achievable [8,9]. Table 1 compares the most relevant public resources. To address this gap, we present SawitMVC.

**Table 1:** Comparison of public datasets for fruit bunch detection and multi-view counting.

Dataset	Crop	Setting	Images	Classes	Annotation	Count †	Multi-view capture
Suharjito et al. (2025) [6]	Oil palm	Plantation	14,503	5	COCO bbox	No	Video (continuous)
Suharjito et al. (2023) [5]	Oil palm	Mill-side piles	7171	6	YOLO bbox	No	Video (360 deg)
Goh et al. (2025) [7]	Oil palm	Field	400	2	Polygon + 3D	No	No
Gene-Mola et al. (2020) [8]	Apple	Orchard	870	1	2D mask + 3D box	Partial	SfM sequence
Gene-Mola et al. (2021) [10]	Apple	Orchard	5356	1	3D segmentation	Partial‡	SfM sequence
Hirschhausen et al. (2025) [9]	Apple	Orchard	9317	1 + 6	Bbox	Yes	Stereo multi-angle
Baglat et al. (2025) [11]	Banana	Field	4864	2–3	Bbox + image-level	No	No
**SawitMVC (ours)**	Oil palm	Plantation	3992	4	YOLO bbox + JSON GT	Yes	Fixed positions (4–8)

† Count GT denotes a per-tree or per-scene unique object count provided independently of detection output.

• Partial indicates that unique fruit identity is inferred from 3D reconstruction rather than explicit expert annotation.

The two rightmost columns set SawitMVC apart. Prior oil palm datasets label bunches for detection and ripeness grading but report no per-tree count that is independent of detection output, so counting accuracy cannot be measured without absorbing detection error. The apple datasets do supply counts, yet they recover bunch identity from structure-from-motion or stereo reconstruction rather than expert annotation. SawitMVC is the only resource that combines per-image bounding boxes, expert per-tree counts, and explicit cross-view identity links from ordinary smartphone photographs. Because these datasets differ in crop, acquisition protocol, annotation target, and evaluation task, a direct numerical comparison of model performance across them would not be meaningful; the comparison here is therefore one of verifiable dataset capability and task coverage.

## 3 Data description

### 3.1 Repository structure

The repository contains four directories and nine root-level files.



The images/ directory holds 3992 JPEG photographs. The labels/ directory holds one annotation file per image in YOLO bounding box format. The json/ directory holds 953 per-tree JSON ground truth files, one per tree. The data/ directory holds a single Parquet file (ground_truth.parquet) that aggregates the per-tree ground truth into a queryable tabular format. Root-level files include data.yaml (YOLO configuration), split manifests (train.txt, val.txt, test.txt), ML Croissant metadata (croissant.json), a download helper (download.py), two annotated sample composites, and README.md. The split manifests define three partitions: 716 trees (3000 images) for training, 96 trees (404 images) for validation, and 141 trees (588 images) for test.

### 3.2 Image files

**Fig. 1:**
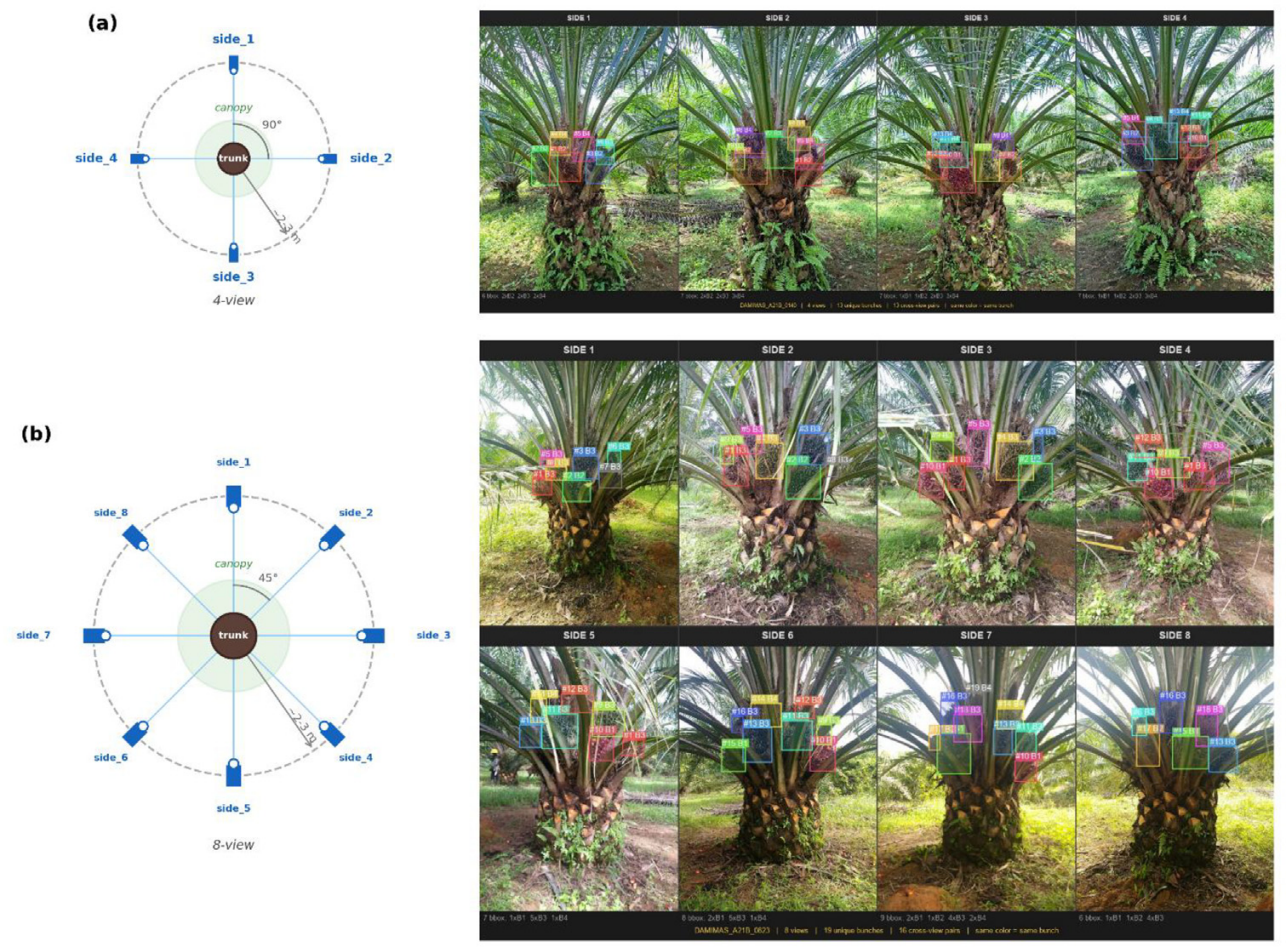
Example annotated trees. (a) A four-view tree (DAMIMAS_A21B_0140), views 1–4. (b) An eight-view tree (DAMIMAS_A21B_0823), views 1–8.

Images in images/ are stored at 960 × 1280 pixels in portrait orientation in JPEG format. The file name encodes tree identity and view index: {VARIETY}_{BLOCK}_{TREEID}_{VIEW}.jpg. For example, DAMIMAS_A21B_0001_1.jpg identifies variety DAMIMAS, field block A21B, tree 0001, and view 1. The view index runs from 1 to 4 for trees photographed at four positions and from 1 to 8 for trees photographed at eight positions. Of the 953 trees, 908 (95.3%) have four views and 45 (4.7%) have eight views. The two root-level sample composites illustrate a complete four-view and a complete eight-view tree (Fig. 1).

### 3.3 Bounding box annotations

The labels/ directory contains one text file per image. Each line encodes one fruit bunch detection in YOLO format: class_id cx cy w h, where class_id is an integer and the spatial values are normalised to image dimensions. The class mapping defined in data.yaml follows the BBC maturity scheme: 0 = B1 (≤1 month to harvest), 1 = B2 (∼2 months), 2 = B3 (∼3 months), 3 = B4 (∼4 months). An example label file line for a B3 bunch:

2 0.4312 0.5781 0.2108 0.3124.

**Fig. 2:**
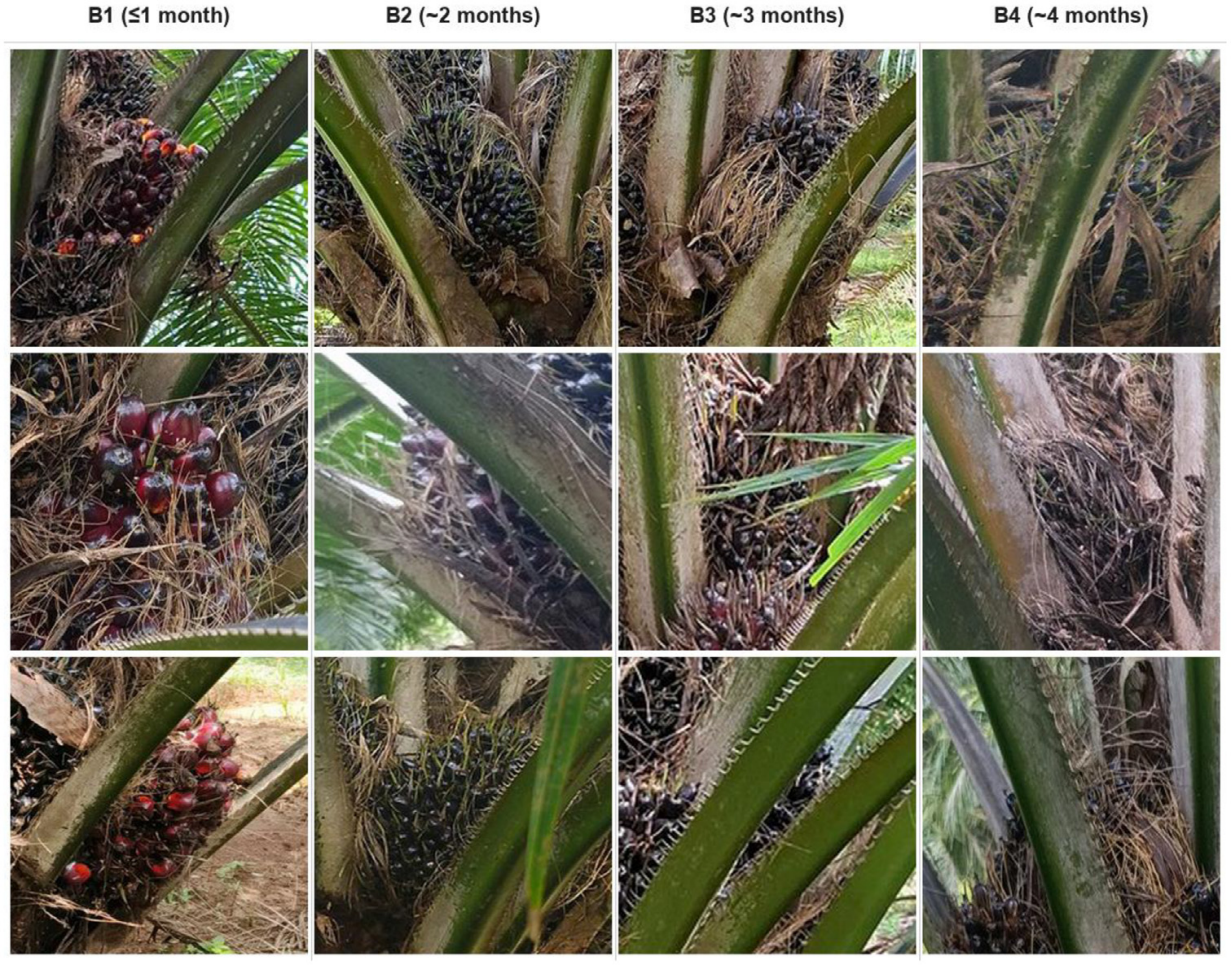
Representative fruit bunch crops for each BBC maturity class.

**Fig. 3:**
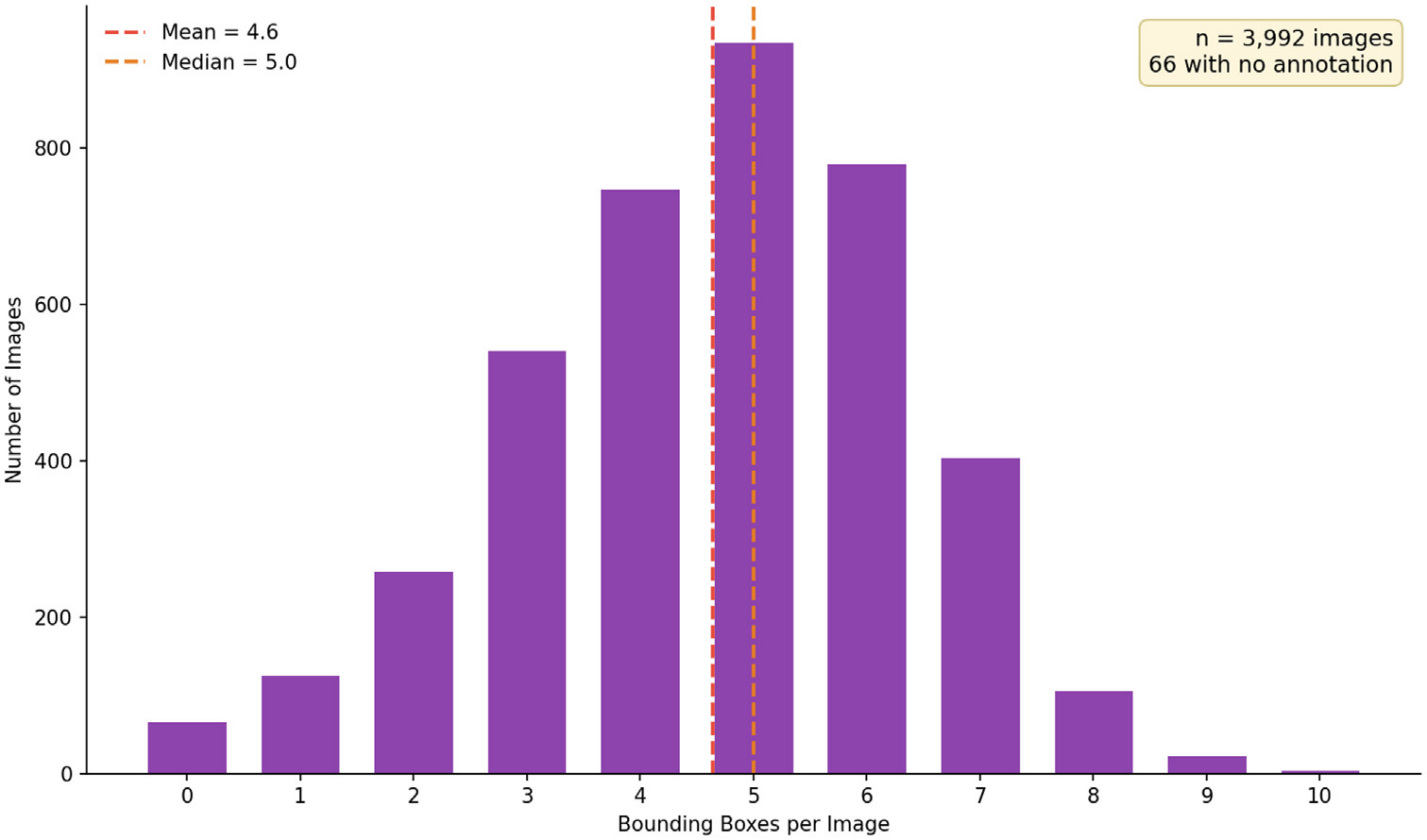
Bounding boxes per image.

Annotators assigned classes based on visual indicators that correlate with development stage. B1 bunches are the largest and most visually distinct, displaying a red-orange colour at the point of optimal harvest. B2 bunches have reached full structural density but retain a dark purple-to-black hue as fruitlets enter the oil accumulation phase. B3 bunches are still expanding volumetrically, presenting tightly packed, elongated black fruitlets. B4 bunches are the smallest, having only recently transitioned from the hidden pre-visible stage. Representative image crops for all four classes are shown in Fig. 2. The dataset contains 18,540 bounding box annotations across 3926 annotated images; 66 images carry zero annotations corresponding to views in which no bunch was visible. Per-image annotation counts range from 0 to 10, with a mean of 4.64 and a median of 5 (Fig. 3).

### 3.4 Ground truth files

The json/ directory contains 953 per-tree JSON files, named after the corresponding tree (e.g., DAMIMAS_A21B_0001.json). Each file stores the tree identifier, variety, and split assignment at the top level, followed by an images object indexing per-view annotation records, a _confirmedLinks array recording pairwise cross-view bunch associations, and a bunches array recording unique bunch identity with maturity class, appearance_count, and view-level cross-references. A minimal top-level structure is shown below:

**Table:** 

{
"version": 4,
"tree_id": "DAMIMAS_A21B_0001",
"split": "train",
"metadata": {
"date": "2026-05-16",
"variety": "DAMIMAS"
},
"images": {
"side_1": {
"filename": "DAMIMAS_A21B_0001_1.jpg",
"side_index": 0,
"side_label": "Side 1",
"bbox_count": 5,
"annotations": [
{
"box_index": 0,
"class_id": 2,
"class_name": "B3",
"bbox_yolo": [0.660417, 0.408203, 0.056250, 0.041406]
}
]
},
"side_2": {...},
"side_3": {...},
"side_4": {...}
},
"bunches": [
{
"bunch_id": 1,
"class": "B3",
"appearance_count": 2,
"appearances": [
{"side": "side_1", "side_index": 0, "box_index": 0},
{"side": "side_4", "side_index": 3, "box_index": 1}
]
}
],
"summary": {
"total_unique_bunches": 8,
"total_detections": 17,
"duplicates_linked": 9,
"by_class": {"B1": 1, "B2": 2, "B3": 5, "B4": 0},
"by_side": {"side_1": 5, "side_2": 4, "side_3": 4, "side_4": 4}
}
}

The metadata.date field records when each per-tree JSON file was finalised through annotation and quality-control sign-off. Photographic capture took place in February 2026, as stated in Section 4.2. Annotation, cross-view linking, and review then ran from March through May 2026. The date stored in each file is therefore the sign-off date for that file's final, post-review version. How bunches is derived from _confirmedLinks is described in Section 4.4 and illustrated in Fig. 7. The data/ground_truth.parquet file aggregates all 9823 unique bunches into a single table with one row per bunch, retaining tree identity, split, variety, class, and appearance count.

### 3.5 Annotation distributions

**Fig. 4:**
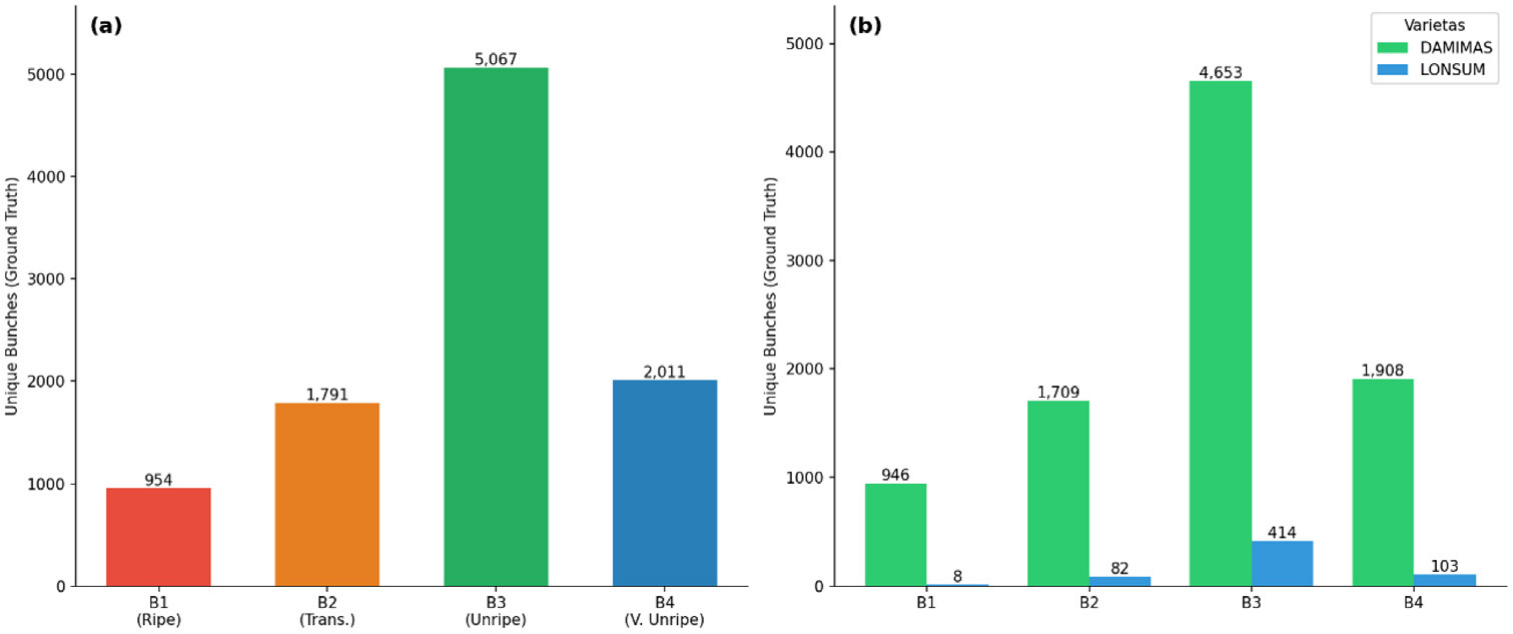
Class distribution (overall + by variety).

**Fig. 5:**
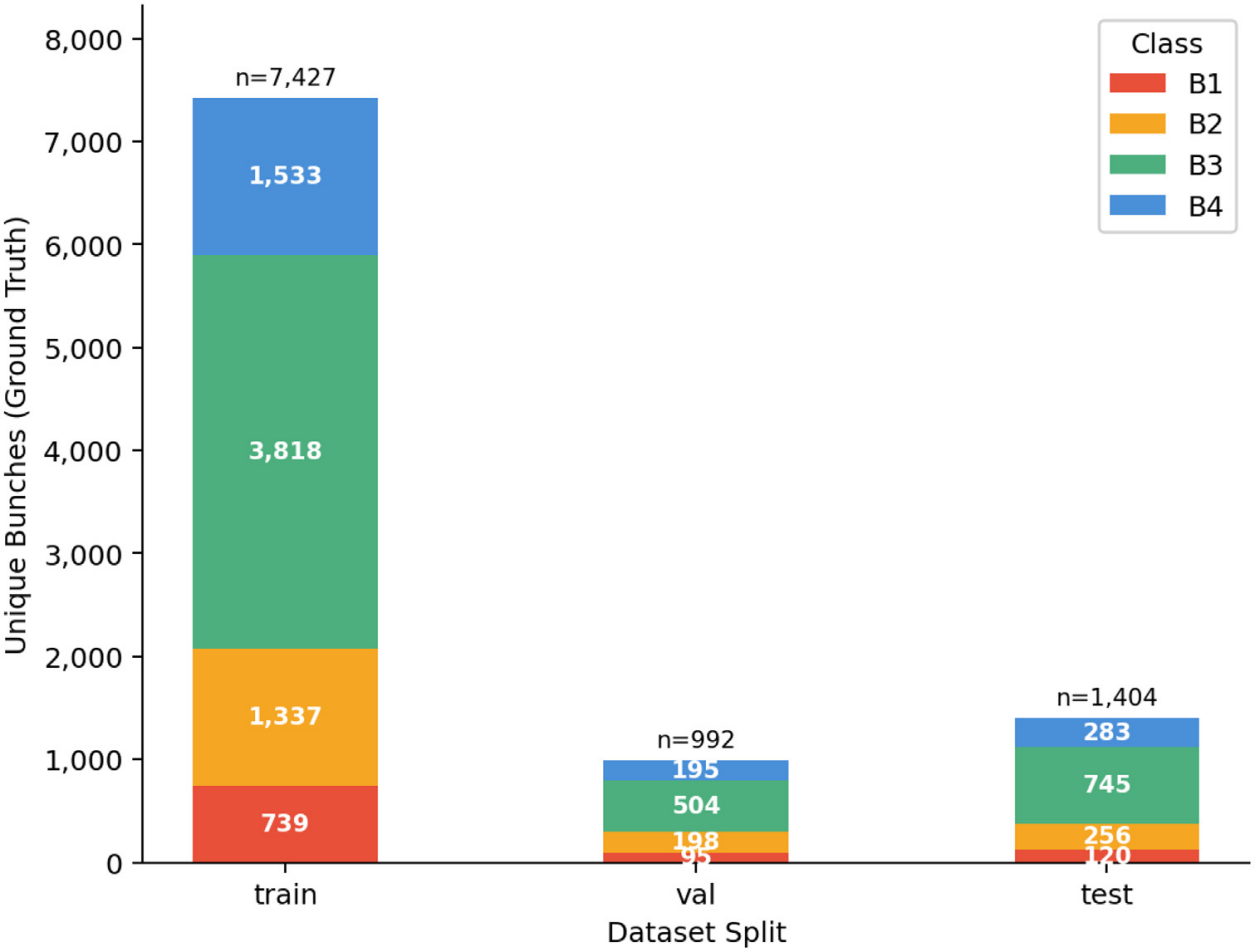
Class distribution by split.

**Fig. 6:**
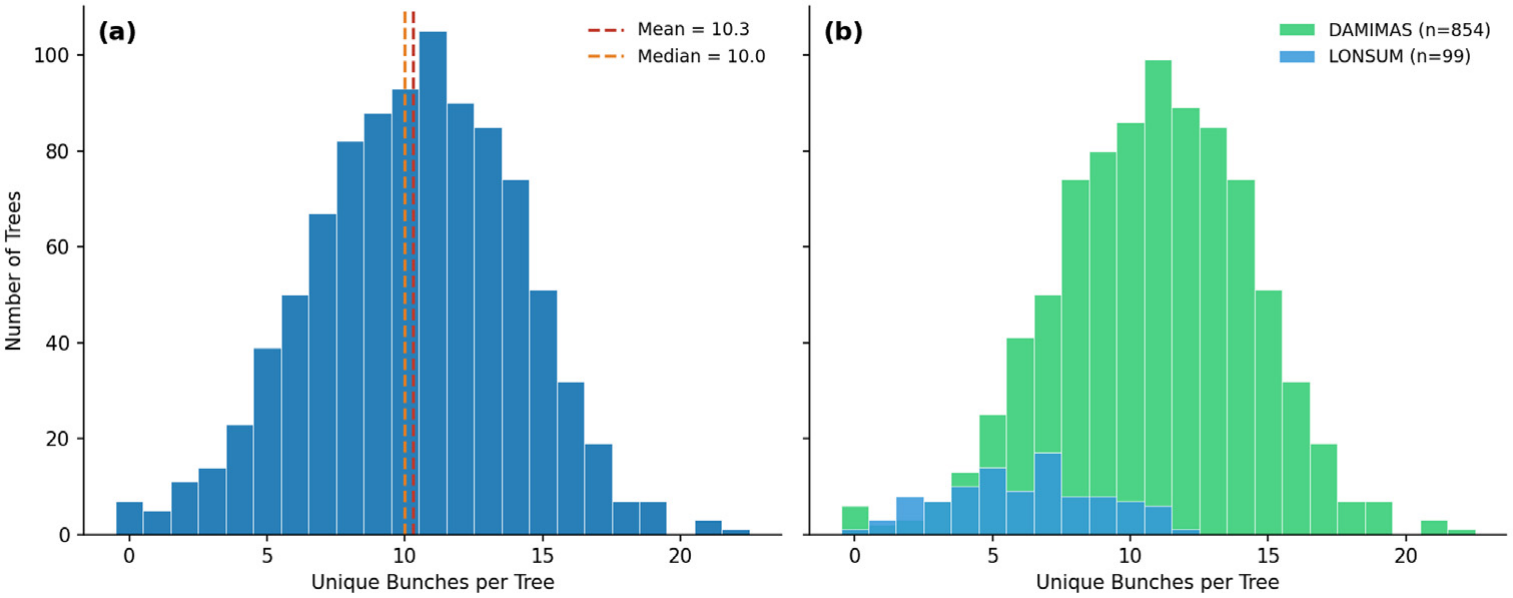
Unique bunch count per tree.

**Fig. 7:**
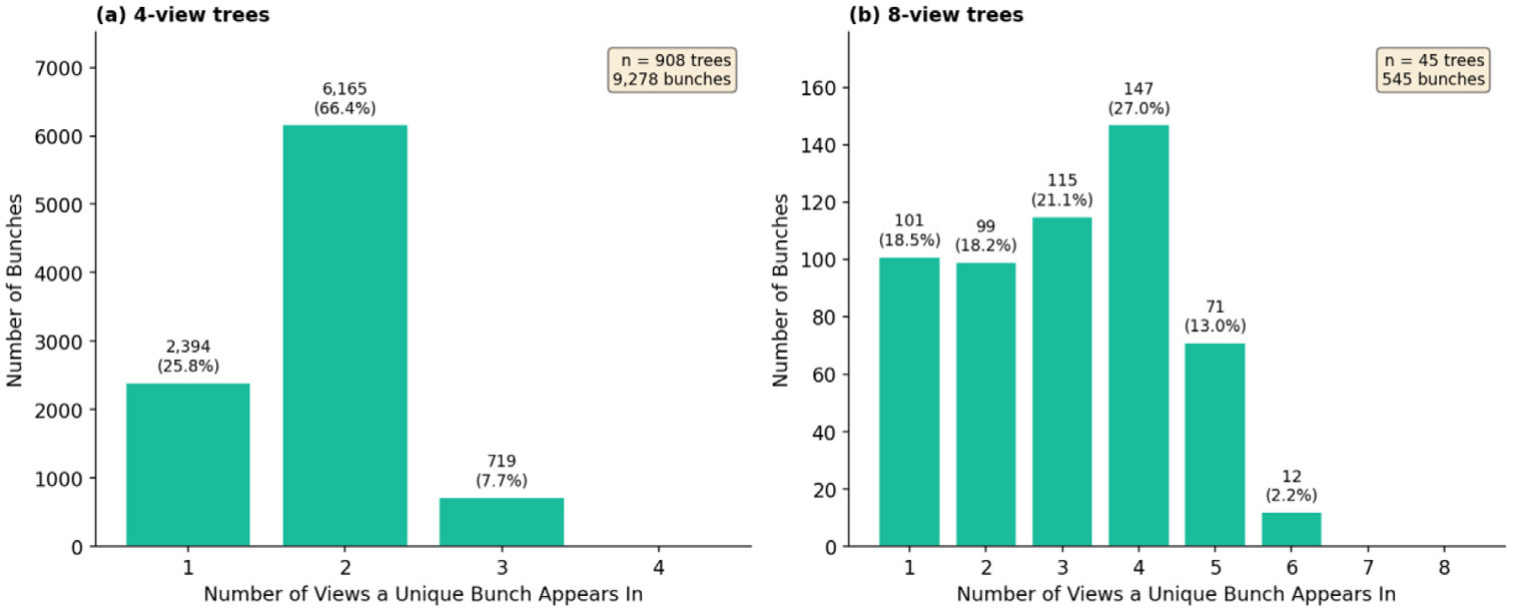
Cross-view visibility of unique fruit bunches for (a) 4-view and (b) 8-view trees.

The 9823 unique bunches are distributed across four maturity classes: B3 accounts for 51.6% (5067 bunches), followed by B4 (20.5%, 2011 bunches), B2 (18.2%, 1791 bunches), and B1 (9.7%, 954 bunches). Class distributions by variety and by split are shown in Fig. 4, Fig. 5. The unique bunch count per tree ranges from 0 to 22, with a mean of 10.31 and a median of 10 (Fig. 6). Each unique bunch appears in 1 to 6 views; 74.6% appear in two or more views, with a mean appearance count of 1.89. The distribution of per-bunch view counts is shown in Fig. 7. Class and annotation counts by split are reported in Table 2.

**Table 2:** Annotation summary by split.

Split	Trees	Images	YOLO Boxes	Unique Bunches	B1	B2	B3	B4
Train	716	3000	14,041	7427	739	1337	3818	1533
Validation	96	404	1887	992	95	198	504	195
Test	141	588	2612	1404	120	256	745	283
Total	953	3992	18,540	9823	954	1791	5067	2011

## 4 Experimental Design, Materials and Methods

### 4.1 Study site and tree selection

Images were collected from two commercial oil palm plantations in Kabupaten Tanah Laut, Kalimantan Selatan, Indonesia in February 2026, identified by their grower-variety label as DAMIMAS plantation (DxP Dami Mas variety, Dami Mas Sejahtera) contributing 854 trees, and LONSUM plantation (PT PP London Sumatra Indonesia variety) contributing 99 trees, for a total of 953 trees across multiple field blocks. Multiple field teams collected data concurrently across assigned areas of each plantation. All collected trees are included in the dataset without post-collection filtering. This includes trees for which no bunches were annotated, either because no bunch was visible from the captured positions or because no bunch was present at the time of collection.

### 4.2 Multi-view image acquisition

Each tree was photographed from four to eight positions spaced at approximately equal angular intervals around the trunk: 90-degree intervals for four-view trees and 45-degree intervals for eight-view trees (Fig. 1). Field operators were instructed to photograph from approximately 2–3 m from the trunk; exact adherence was not verified. Images were captured using ten consumer smartphone models: Xiaomi Redmi Note 12 Pro 5 G, Redmi Note 11 Pro 5 G, and Poco F6; Samsung Galaxy A55 5 G, A52 5 G, and A56 5 G; Apple iPhone 11 and iPhone 14 Pro; Realme C33; and Infinix Hot 10 s. All images were stored in JPEG format at 960 × 1280 pixels in portrait orientation. Exposure settings were left to each device's automatic mode.

A custom mobile application (PohonKu) was used by field operators to manage tree registration, view sequencing, and metadata capture. PohonKu assigned each tree a unique identifier encoding variety and field block (e.g., DAMIMAS_A21B), numbered trees sequentially within each block, and recorded the view index for each photograph. Images were named following the convention {VARIETY}_{BLOCK}_{TREEID}_{VIEW}.jpg. Of the 953 trees, 908 (95.3%) were photographed from four positions and 45 (4.7%) from eight positions.

### 4.3 Bounding box annotation

Bounding box annotations were applied to all 3992 images using a custom web-based annotation tool developed for this project. Seven trained annotators drew a rectangular bounding box around each visible fruit bunch and assigned one of four maturity class labels (B1–B4, defined in Section 3.3). Class definitions followed agronomic maturity criteria verified by an expert agronomist, who supervised the annotation process throughout. Completed annotations were reviewed in full by a single reviewer, who applied corrections before export. Annotations were exported in YOLO bounding box format as described in Section 3.3.

### 4.4 Cross-view bunch identity annotation

Following bounding box annotation, cross-view bunch identity links were established using the same custom web-based annotation tool. The tool presented two adjacent views of the same tree side by side. Annotators identified bounding boxes in each view that corresponded to the same physical bunch and recorded a pairwise confirmed link. Each link is stored in the per-tree JSON file under the _confirmedLinks array, with fields sideA, bboxIdA, sideB, and bboxIdB identifying the two connected bounding boxes by their side index and box index within that side. Progressing through the four or eight sides of each tree, annotators produced a set of pairwise links forming a graph over all bounding boxes for that tree.

**Fig. 8:**
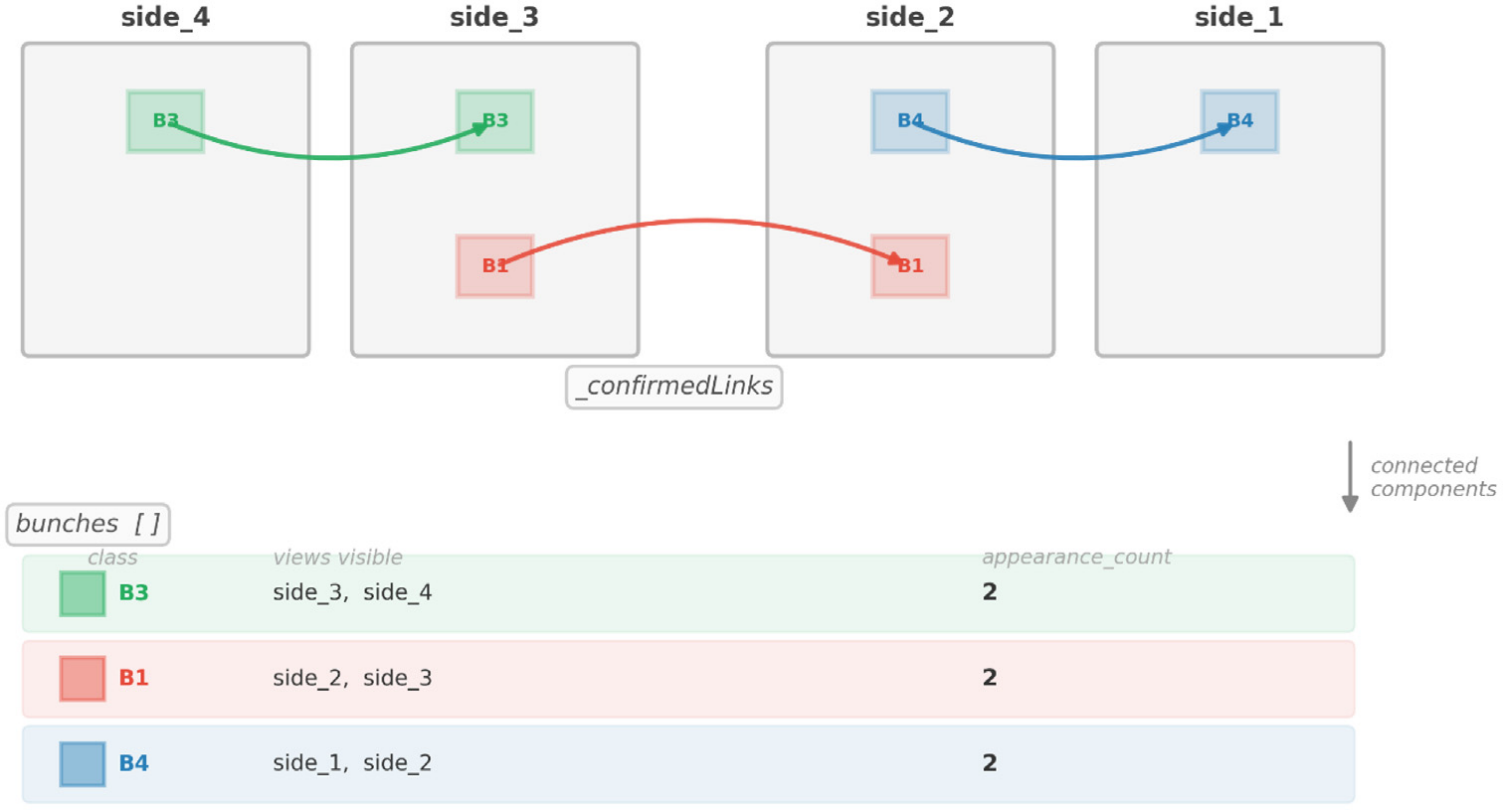
Cross-view bunch identity linking scheme.

Unique bunch identity was derived by computing connected components (transitive closure) of the _confirmedLinks graph. Each connected component corresponds to one unique physical bunch. The resulting bunches are stored in the bunches array of the per-tree JSON file. Each bunch record lists its assigned maturity class, appearance_count (number of views in which it is visible), and an appearances array cross-referencing the exact bounding box in each view by side label, box index, and pixel-space coordinates. A class_mismatch flag is set automatically during this derivation step when the annotated class differs across views within the same connected component. The linking scheme is illustrated in Fig. 8.

### 4.5 Train, validation, and test split

Trees were partitioned into training (716 trees), validation (96 trees), and test (141 trees), in an approximately 75/10/15 ratio by tree count, with stratification by variety and by dominant maturity class, so that each split preserves the overall distribution of both factors. All images of a given tree were assigned to the same split. The split assignment for each tree is recorded in the split field of its JSON file and in the ground_truth.parquet summary table. Image paths for each split are listed in train.txt, val.txt, and test.txt. Split counts and per-class annotation totals are reported in Table 2.

### 4.6 Ground truth summary and dataset packaging

The per-tree JSON files were aggregated into a single Parquet file (data/ground_truth.parquet) with one row per unique bunch, retaining fields for tree identifier, variety, split, maturity class, and appearance count. The Parquet format supports efficient querying and is compatible with standard data analysis libraries. ML Croissant metadata (croissant.json) describes the dataset structure and field semantics in a machine-readable format to support dataset discovery and loading via compatible frameworks. The dataset is released on Zenodo under the Creative Commons Attribution-NonCommercial 4.0 International (CC BY-NC 4.0) licence.

### 4.7 Baseline experiments

The dataset supports two evaluation targets, detection and counting, and a baseline is provided for each. Detection establishes that the bounding box annotations train a working detector; counting establishes that the per-tree ground truth supports cross-view deduplication independently of detection quality. Both are deliberately simple reference points rather than tuned systems, leaving more thorough method development to future work that the dataset is intended to enable.

For detection, YOLO26m was fine-tuned on the 716-tree training split using the provided bounding box annotations, with epochs=60, batch=32, imgsz=640, patience=60, and seed=42. Per-class precision, recall, and AP50 on the held-out test split are reported in Table 3.

**Table 3:** YOLO26m detection results on the test split.

Model	AP50	Precision	Recall
Overall	0.531	0.508	0.571
B1	0.739	0.602	0.776
B2	0.433	0.482	0.441
B3	0.599	0.515	0.674
B4	0.354	0.432	0.393

For counting, two strategies were applied to the per-view detections of each tree and evaluated on the 141-tree test split (Table 4). The first is a global correction factor that divides the naive class-wise sum of detections by k = 1.8905, then rounds to the nearest integer. This divisor is the ratio of total bounding box annotations to unique bunches in the training split (14,041 / 7427). It equals the mean number of views in which a bunch appears, consistent with the dataset-wide mean appearance count of 1.89 reported in Section 3 (18,540 / 9823 = 1.887).

**Table 4:** Bunch counting baselines on the test split (141 trees), under ground-truth and YOLO26m detections.

Detection	Counter	Class ±1 Acc	Tree ±1 Acc	Macro MAE	Mean Bias
Ground truth	Naïve sum	50.00%	6.38%	2.142	+2.142
Ground truth	Global correction (k = 1.89)	95.57%	86.52%	0.356	+0.009
Ground truth	SVR	96.81%	88.65%	0.303	−0.048
YOLO26m	Global correction (k = 1.89)	72.34%	30.50%	1.119	0.381
YOLO26m	SVR	75.35%	33.33%	1.027	0.158

a) Class ±1 Acc: fraction of trees where the predicted count falls within ±1 of the ground truth, macro-averaged across B1–B4.

b) Tree ±1 Acc: fraction of trees where all four classes are simultaneously within ±1.

c) Macro MAE and Mean Bias: per-class mean absolute error and signed error (predicted − true), macro-averaged across B1–B4.

Dividing by k rescales the inflated multi-view sum by the average degree of cross-view duplication, which makes it a natural heuristic deduplication reference. Because k is estimated on the training split alone, it carries no test-set information.

The second strategy is support vector regression (SVR), used as a learned baseline that predicts per-class unique counts from a 13-dimensional cross-view feature vector. These features summarise the cross-view detection pattern of each tree: the naive per-class sum, the maximum and mean per-class count across individual sides, and the number of sides photographed. Unlike the single-ratio divisor, SVR can learn class-specific and view-count-dependent deduplication behaviour.

Four independent SVR models were trained, one per maturity class (B1 to B4), since the implementation uses single-output regressors. Each model used a radial basis function kernel with scikit-learn default settings (C = 1.0, epsilon = 0.1, gamma = "scale"); no hyperparameter search or validation-split selection was performed, so the counter is a deliberately untuned reference. Features were passed without standardisation, and predictions were clipped at zero and rounded to the nearest integer before computing the count metrics.

Each strategy was evaluated under two detection conditions. The ground-truth condition feeds annotated detections to the counter, which isolates counting quality from detector error and establishes an upper reference. The YOLO26m condition feeds the fine-tuned detector's predictions and reports end-to-end performance obtainable from the released annotations under the detector configuration described above.

SVR improves on the correction factor under both conditions, most clearly on the stricter Tree ±1 accuracy and on MAE. The gap between conditions is, however, large: test Class ±1 accuracy falls from 96.81% under ground-truth detections to 75.35% under YOLO26m. Detection, rather than the counting stage, is therefore the dominant source of error in the end-to-end pipeline, a question the dataset is positioned to support.

## Limitations

All images were collected from two commercial plantations in Kalimantan, Indonesia during a single collection period in February 2026. The dataset therefore does not capture seasonal changes in bunch maturity distribution, or wider plantation management differences that may affect appearance and class proportions in other settings.

Two commercial varieties are represented (DAMIMAS and LONSUM), with DAMIMAS comprising 854 of 953 trees (89.6%). Performance of models trained on this dataset may not generalise to other commercial varieties not present in the collection.

Images were captured using different consumer smartphone models under ambient outdoor light with automatic exposure. No controlled lighting or depth information was recorded. Image quality varies across devices and lighting conditions. Future extensions could incorporate depth or reconstructed 3D geometry, but the present release intentionally targets an RGB-only workflow deployable with ordinary consumer smartphones.

Maturity class labels carry irreducible ambiguity at the B2/B3 boundary, where visual distinction between transitioning and unripe bunches is uncertain even under expert supervision. No formal inter-annotator agreement was measured.

No per-tree metadata such as tree age or canopy height was recorded.

## Ethics Statement

This work does not involve human subjects, animal experiments, or any data collected from social media platforms. The authors have read and follow the ethical requirements for publication in Data in Brief.

## CRediT Author Statement

**Fatma Indriani:** Conceptualization, Funding acquisition, Methodology, Project administration, Supervision, Investigation, Formal analysis, Writing – original draft; **Setyo Wahyu Saputro:** Methodology, Investigation, Resources, Formal analysis, Software, Writing – review & editing; **Muhammad Zainal Muttaqin:** Investigation, Data curation, Formal analysis, Software, Writing – review & editing; **Alia Rahmi:** Data curation, Investigation, Validation, Software, Writing – review & editing; **Triando Hamonangan Saragih:** Investigation, Formal analysis, Software, Writing – review & editing; **Rahmat Budianoor:** Software; **Hartoni:** Validation; **Dwi Kartini:** Investigation; **Naufal Said:** Investigation.

## Data Availability

ZenodoSawitMVC (Original data).
